# Evolution of Surgical Approaches for Trigeminal Schwannomas: A Meta-Regression Analysis from Past to Present

**DOI:** 10.3390/jcm14134488

**Published:** 2025-06-25

**Authors:** Edoardo Porto, Giorgio Fiore, Cecilia Casali, Mario Stanziano, Morgan Broggi, Giulio A. Bertani, Hani J. Marcus, Marco Locatelli, Francesco DiMeco

**Affiliations:** 1 Department of Neurosurgery, Fondazione IRCCS Istituto Neurologico Carlo Besta, 20133 Milan, Italy; cecilia.casali@istituto-besta.it (C.C.); morgan.broggi@istituto-besta.it (M.B.); francesco.dimeco@istituto-besta.it (F.D.); 2 Department of Neurosurgery, Emory University, Atlanta, GA 30322, USA; 3 Unit of Neurosurgery, Fondazione IRCCS Ca’ Granda Ospedale Maggiore Policlinico, 20122 Milan, Italy; giulio.bertani@policlinico.mi.it (G.A.B.); marco.locatelli@policlinico.mi.it (M.L.); 4 Department of Clinical and Experimental Epilepsy, UCL Queen Square Institute of Neurology, University College London, London WC1N 3BG, UK; 5 Neuroradiology Unit, Diagnostic and Technology Department, Fondazione IRCCS Istituto Neurologico Carlo Besta, 20133 Milan, Italy; mario.stanziano@istituto-besta.it; 6 ALS Centre, “Rita Levi Montalcini” Department of Neuroscience, University of Turin, 10126 Turin, Italy; 7 Victor Horsley Department of Neurosurgery, National Hospital for Neurology and Neurosurgery, London WC1N 3BG, UK; hani.marcus@nhs.net; 8 Department of Pathophysiology and Transplantation, University of Milan, 20122 Milan, Italy; 9 Department of Neurosurgery, Johns Hopkins University, Baltimore, MD 21218, USA

**Keywords:** trigeminal schwannoma, trigeminal neurinoma, ETOA, transorbital approach, EEA, subtemporal approach, trigeminal neuralgia, trigeminal dysesthesia

## Abstract

**Background/Objectives:** The surgical management of trigeminal schwannomas (TSs) has evolved considerably, with increasing interest in minimally invasive approaches. We performed a meta-regression analysis to characterise temporal trends in surgical strategies for TS and to explore factors influencing outcomes. **Methods:** This systematic review and meta-regression followed the PRISMA 2020 guidelines. Comparative studies published in English reporting surgical treatment of TS were included. Outcomes assessed were the extent of resection (EOR), improvement or worsening of trigeminal symptoms, and postoperative complications. Meta-analyses of pooled frequencies were performed, and meta-regression analyses evaluated associations between surgical approach, tumour localization, year of publication, and outcomes. Surgical approaches were categorized as microsurgical antero-lateral (M-AL-Apr), retrosigmoid (RSA), endoscopic endonasal (EEA), and endoscopic transorbital (ETOA). Tumour localization was stratified using the Samii classification. **Results:** Fifteen studies (583 surgeries) were included. Endoscopic approaches accounted for 20.1% of cases, with increasing use over time (β = 0.12—*p* < 0.001), largely driven by transorbital access for Samii type A and C tumours. The use of M-AL-Apr declined. The pooled gross-total resection (GTR) rate was 73% (I^2^ = 78.8%). The stratified meta-regression identified a temporal decrease in GTR for Samii type C tumours alone, while resection rates for types A, B, and D remained stable, likely reflecting the increasing proportion of anatomically complex cases in recent series Trigeminal impairment improved postoperatively in 17% (I^2^ = 84.5%), while worsening of trigeminal symptoms was rare (β = 0.07%—I^2^ = 0%). Complication rates were 11.6% (I^2^ = 32.7%) but with a temporal increase (β = 0.041, *p* = 0.047). Tumour type was the dominant predictor of EOR, functional outcomes, and complications. **Conclusions:** Surgical management of TS has evolved towards minimally invasive techniques, particularly endoscopic routes, reflecting advances in technology and a focus on functional preservation. Tumour anatomy remains the key determinant of surgical outcomes, highlighting the importance of tailored, anatomy-driven surgical planning.

## 1. Introduction

**Figure 1:**
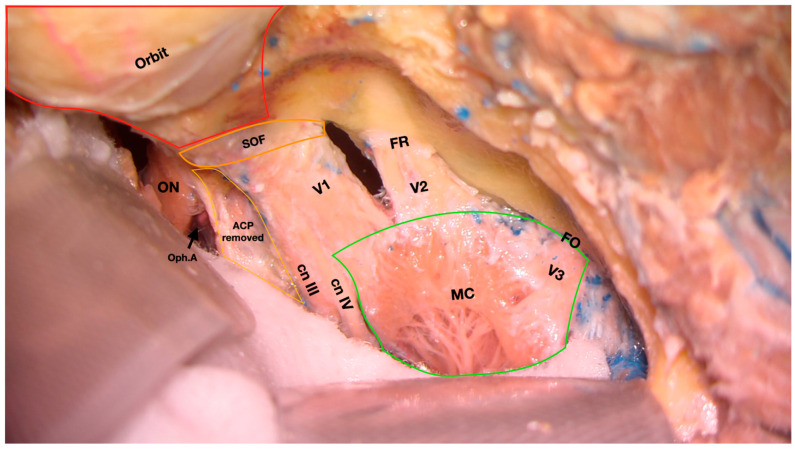
Cadaveric dissection showing the middle cranial fossa following removal of the superior and lateral walls of the right orbit. Meckel’s cave is exposed following the removal of the periosteal layer of the dura mater. ON, optic nerve; Oph.A, ophthalmic artery; ACP, anterior clinoid process; cn III, cranial nerve III; cn IV, cranial nerve IV; V1, first branch of the trigeminal nerve; V2, second branch of the trigeminal nerve; V3, third branch of the trigeminal nerve; FR, foramen rotundum; FO, foramen ovale. SOF, superior orbital fissure; MC, Meckel’s cave. (First author’s dissection).

Trigeminal schwannomas (TS) are rare, typically benign tumours originating from the Schwann cells of the fifth cranial nerve. Accounting for approximately 0.8% to 8% of all intracranial schwannomas, these lesions can arise along any portion of the trigeminal pathway, from the root entry zone at the brainstem to the extracranial peripheral branches [1]. (Figure 1) Due to their anatomical variability and frequent proximity to critical neurovascular structures of the skull base, surgical resection remains complex. Although stereotactic radiosurgery may be an option for selected small or asymptomatic tumours, surgical resection is often the treatment of choice for symptomatic or enlarging lesions [2].

Historically, TSs have been managed through traditional craniotomy-based approaches, including posterior routes (retrosigmoid), and lateral approaches such as subtemporal, frontotemporal (pterional), and fronto-orbitozygomatic (FOZ) variants [3]. These techniques offer wide exposure and the ability to manage tumours involving the middle and posterior cranial fossae. However, they often require significant brain retraction and manipulation of surrounding structures, with associated risks of postoperative morbidity. Despite refinements in microsurgical technique, these approaches are inherently invasive and may not be optimal for all tumour locations or patient profiles [4].

In recent years, minimally invasive strategies have gained increasing attention, particularly the endoscopic endonasal (EEA) and endoscopic transorbital (ETOA) approaches. While the EEA route offers a direct midline trajectory to lesions involving Meckel’s cave and the central skull base, the ETOA has emerged as a versatile and promising option for lesions involving the antero-lateral skull base, cavernous sinus, and middle fossa [5]. The ETOA route, in particular, allows for lateral access with minimal brain retraction and improved cosmetic outcomes, making it an increasingly attractive alternative or complement to conventional approaches [6,7,8]. Nonetheless, both endoscopic techniques come with their own set of challenges, including restricted working corridors, demanding reconstruction, and a steep learning curve [9]. Comparative data on indications, the extent of resection, functional outcomes, and complication rates are limited.

This systematic review and meta-analysis primarily aimed to investigate whether a shift in the surgical management strategy of TSs has occurred over time. Specifically, it assessed temporal trends in the adoption of various surgical approaches, including both traditional open craniotomies and more recent endoscopic techniques. Secondarily, the study evaluated whether different treatment strategies over time have resulted in variations in the extent of resection (EOR), complication rates, and functional outcomes. By synthesizing current evidence, this analysis seeks to clarify the evolving role of each surgical corridor and guide decision-making based on anatomical, pathological, and temporal considerations (Figure 1).

## 2. Materials and Methods

### 2.1. Study Design and Eligibility Criteria

This systematic review and meta-analysis were conducted in accordance with the PRISMA 2020 guidelines [10].

Eligible studies were observational in design and reported on surgical series of patients with TSs undergoing resection via either traditional open craniotomy approaches (e.g., subtemporal, frontotemporal, fronto-orbitozygomatic [FOZ], or retrosigmoid [RSA]) or endoscopic techniques (EEA or ETOA). Studies were required to provide information on at least one of the outcomes of interest and to report the surgical approach used.

**Figure 2:**
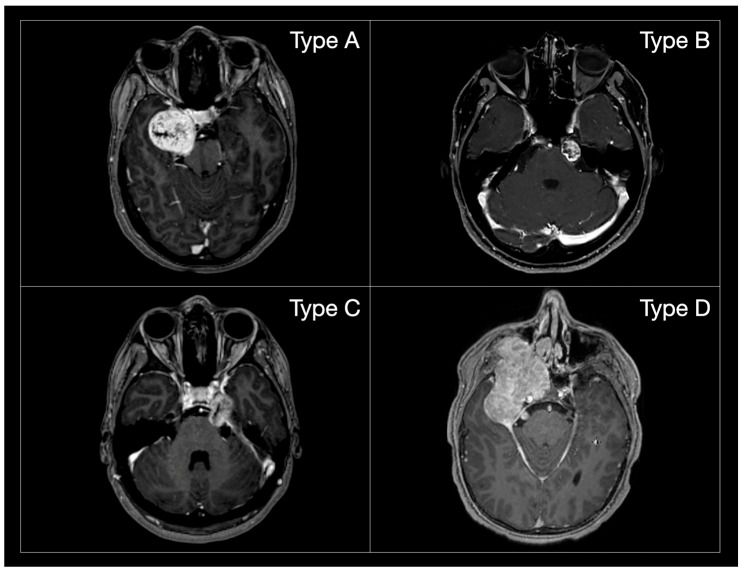
MR post-gadolinium T1 axial images of the different Samii TS subtypes. (**Type A**): tumours mainly extending in the middle cranial fossa; (**Type B):** tumours mainly extending in the posterior cranial fossa; (**Type C**): dumbbell tumours; (**Type D**): tumours extending in the extra-cranial compartment.

To ensure consistency in anatomical comparison, tumour localization was classified according to the Samii classification, which stratifies TSs into four types based on their anatomical extension: Type A (middle fossa), Type B (posterior fossa), Type C (dumbbell-shaped, involving both compartments) and Type D (extracranial extension) [11] (Figure 2).

No restrictions were placed on the study period or language, provided that an English version was available. Case reports and small case series (less than 10 cases), reviews, editorials, and conference abstracts were excluded.

### 2.2. Literature Search and Study Selection

A comprehensive literature search was conducted across MEDLINE (PubMed), Embase, Scopus, and Web of Science, using combinations of the following MeSH terms and free-text keywords: “trigeminal schwannoma”, “trigeminal nerve tumour”, “skull base surgery”, “endoscopic endonasal”, “transorbital”, “craniotomy”, “retrosigmoid”, “subtemporal”, “fronto-orbitozygomatic”, and “surgical outcomes”. References of included studies were also screened to identify additional eligible articles. The final search update was performed in May 2025.

### 2.3. Data Extraction

Two authors (EP and GF) independently screened titles, abstracts, and full texts for inclusion. Any discrepancies were resolved by consensus. Data extraction was also performed independently by the same reviewers, using a predefined template. Extracted variables included study characteristics (author, year of publication, number of patients), tumour distribution according to Samii classification, the surgical approach used (EEA, ETOA, RSA, and microsurgical antero-lateral approaches [M-AL-Apr] [such as pre- and subtemporal, FOZ, and Kawase approaches]), and surgical outcomes.

### 2.4. Outcomes

The primary outcomes were EOR (gross-total [GTR] and subtotal [STR]), the presence of trigeminal impairment (hypoesthesia, dysesthesia, or facial pain) before and after surgery, changes in trigeminal symptoms (either improvement or worsening), and the rate of postoperative complications (cranial nerve deficits, CSF leak, infection, vascular injury, brain-retraction-related complications).

In order to explore whether surgical practice and outcomes have changed over time and whether they are influenced by tumour type or surgical approach, we constructed a comprehensive dataset that included the proportion of patients treated with each surgical approach, as well as the distribution of Samii tumour types within each study. We also quantified the percentage of each Samii tumour type over time and visualized their temporal evolution using an alluvial diagram to assess whether more challenging tumour subtypes have become more commonly treated in recent years.

### 2.5. Statistical Analysis

For each outcome, pooled incidence rates were estimated using random-effects meta-analysis of proportions (GLMM method). Statistical heterogeneity was quantified using the I^2^ and τ^2^ statistics. Forest plots were used to display the pooled effects, the confidence intervals (CI), and the estimated measures of inter-study heterogeneity.

To investigate whether outcomes varied according to surgical approach, tumour type, or publication year, meta-regression analyses were conducted using binomial generalized linear models (GLMs). Separate univariable models were first fitted to assess the influence of each predictor individually. Subsequently, multivariable models were constructed incorporating all key variables, namely surgical approach, Samii tumour type, and year of publication. The significance of each factor within the multivariable framework was assessed using likelihood ratio tests.

To explore if any variation in GTR rates was driven by specific tumour subtypes, we performed subgroup analyses stratified by dominant Samii type. For each group, GTR rates were modelled over time using GLMs with year quartile as a categorical variable, and the effect of time was evaluated using likelihood ratio testing.

No imputation was performed. Studies reporting zero events were included in the analyses as such. All statistical analyses were conducted in R software 4.2.2 (R Foundation for Statistical Computing, Vienna, Austria; https://www.r-project.org/ [accessed on 31 May 2025]).

### 2.6. Risk of Bias

Risk of bias was assessed independently by two reviewers (EP and GF). The Joanna Briggs Institute (JBI) checklist for case series and cohort studies was used [12]. Studies scoring ≥ 7 were considered methodologically acceptable. Discrepancies were resolved through consensus.

### 2.7. Assessment of Reporting Bias and Sensitivity Analysis

Influence analyses were conducted to assess whether individual studies disproportionately affected pooled results. Sensitivity analyses excluded studies at high risk of bias or those lacking stratified outcomes.

## 3. Results

### 3.1. Study Selection and Characteristics

**Figure 3:**
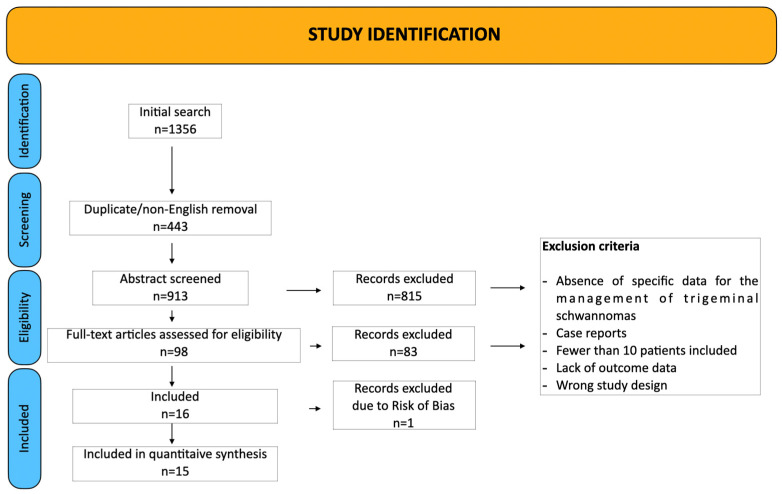
PRISMA chart.

A total of 15 studies was included in this meta-analysis [3,4,6,11,13,14,15,16,17,18,19,20,21,22,23], encompassing 583 patients who underwent surgeries for TS. The studies spanned publication years 1988 to 2025, with cohort sizes ranging from 12 to 96 patients. The PRISMA flow diagram is provided in Figure 3.

The distribution of Samii tumour types was as follows: type A (22%), type B (11%), type C (42%), and type D (25%). Surgical approaches included M-AL Apr (64.2%, n = 374), RSA (11.3%, n = 66), ETOA (10.5%, n = 61), EEA (9.6%, n = 56), combined/two-stage approaches (Comb/2sps; 3.9%, n = 23), and the trans-maxillary approach (TMA; 0.5%, n = 3) (Table 1). Follow-up time was variable across the studies included (48.8 ± 52.6 months).

### 3.2. Choice of Surgical Approaches and Evolution over Time

**Figure 4:**
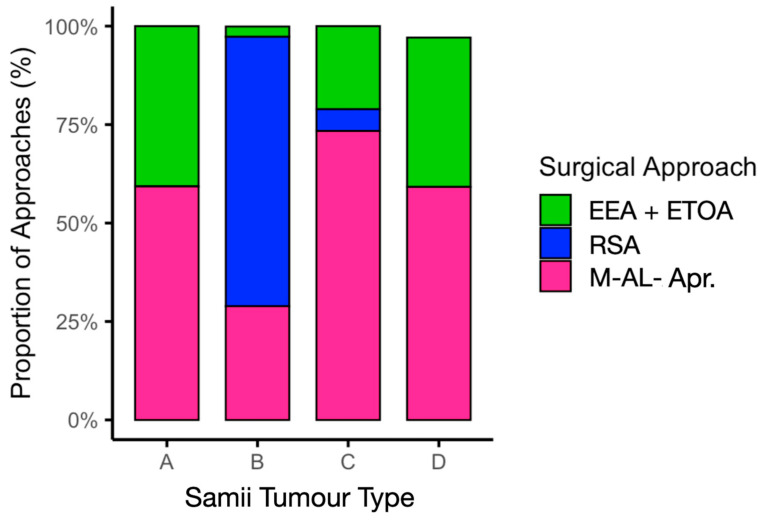
Surgical approach distribution by Samii tumour type. M-AL-Apr, microsurgical antero-lateral approaches.

Meta-regression confirmed that Samii tumour type significantly determined the choice of approach (*p* < 0.001). EEA or ETOA was adopted in 40.7% of Samii type A tumours, 2.6% of type B, 21.1% of type C, and 37.9% of type D. M-AL approaches predominated for type C tumours (73.4%), and RSA was the preferred approach for type B tumours (68.4%). This distribution is illustrated in Figure 4 and Table 2.

**Figure 5:**
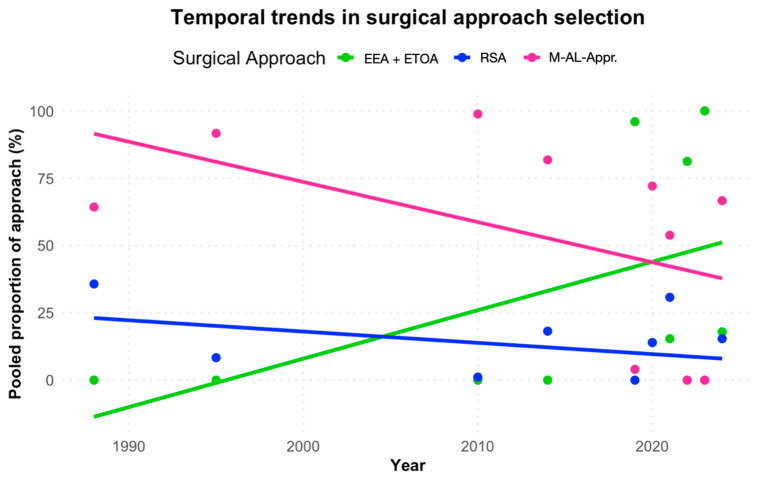
Surgical approach distribution over time. M-AL-Apr, microsurgical antero-lateral approaches.

The use of endoscopic approaches increased significantly over time (β = +0.12, 95% CI 0.07–0.17, *p* < 0.001), corresponding to an estimated +12.7% relative increase per year. In contrast, the use of M-AL-Apr declined significantly (β = −0.13, 95% CI −0.21 to −0.06, *p* < 0.001), corresponding to a −12.2% relative decrease per year. The use of RSA remained stable (β = +0.01, 95% CI −0.02 to +0.04, *p* = 0.47) (Figure 5).

**Figure 6:**
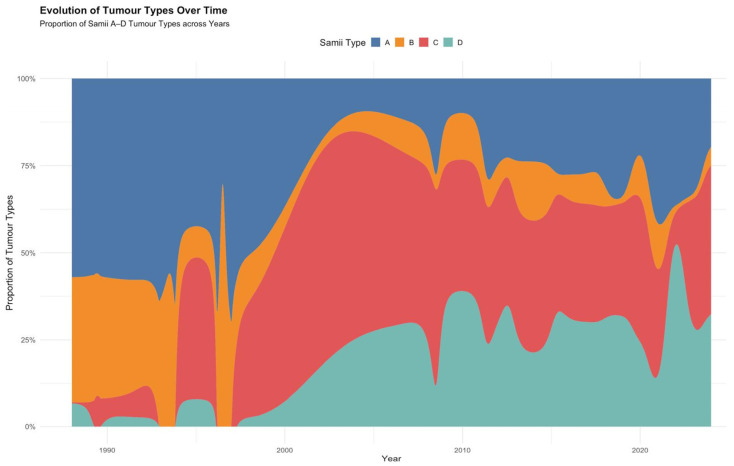
Alluvial diagram showing the evolution of Samii tumour types over time.

To contextualize the shifting surgical strategies, we also examined how the proportion of Samii tumour types evolved across the included studies. The alluvial plot (Figure 6) illustrates a gradual increase in the relative frequency of Samii type C and D tumours over the past three decades, whereas type A tumours declined in more recent years.

### 3.3. Extent of Resection Outcomes

The pooled rate of GTR was 73.2% (95% CI 63.4–81.2%; I^2^ = 78.8%; Figure 6). Meta-regression showed a decrease in GTR rates over time (β = −0.054, 95% CI −0.087 to −0.022, *p* < 0.001), corresponding to a 5.3% relative reduction per year.

In multivariable analysis, RSA use (β = −13.66, *p* < 0.001) and higher proportions of Samii type B (β = −42.33, *p* = 0.010), type C (β = −59.75, *p* = 0.002) and type D tumours (β = −60.30, *p* = 0.002) were independently associated with lower likelihood of achieving GTR (Supplementary Table S1).

To further investigate whether the observed reduction in GTR rates was uniformly distributed across tumour types, we analysed GTR trends stratified by Samii classification. While no significant change was detected for Samii types B and D, and only marginal changes for type A, a marked and statistically significant decline in GTR was observed in Samii type C tumours (*p* < 0.001; Supplementary Table S2).

**Figure 7:**
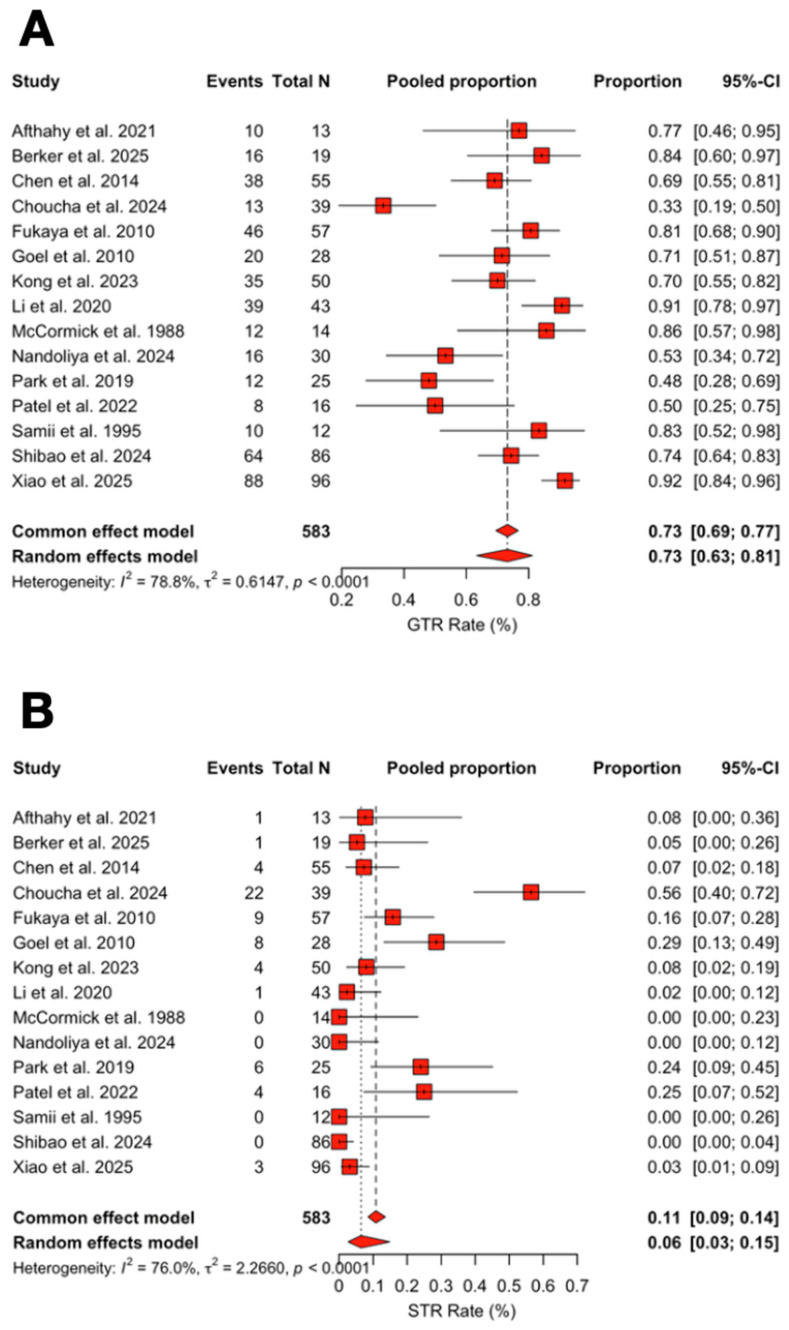
(**A**) Forest plot showing GTR rate across studies included. (**B**) Forest plot showing STR rate across studies included [3,4,6,11,13,14,15,16,17,18,19,20,21,22,23]. Red refers to Extent of Resection rates. The overall meta-analysed measure of effect is represented on the plot as a dashed vertical line. Squares represent individual study effect sizes (with relative solid lines representing confidence intervals). Diamonds summarize the overall pooled effect and its confidence interval.

The pooled rate of subtotal resection (STR) was 6.5% (95% CI 2.7–14.9%; I^2^ = 76.0%). STR rates increased significantly over time (β = +0.067, 95% CI +0.024 to +0.116, *p* = 0.004), corresponding to a 6.9% relative increase per year (Figure 7A,B).

### 3.4. Functional Outcomes

The pooled rate of preoperative trigeminal symptoms was 72.1% (95% CI 61.6–80.6%; I^2^ = 56.4%). The pooled rate of postoperative trigeminal impairment was 46.5% (95% CI 22.9–71.7%; I^2^ = 75.3%).

**Figure 8:**
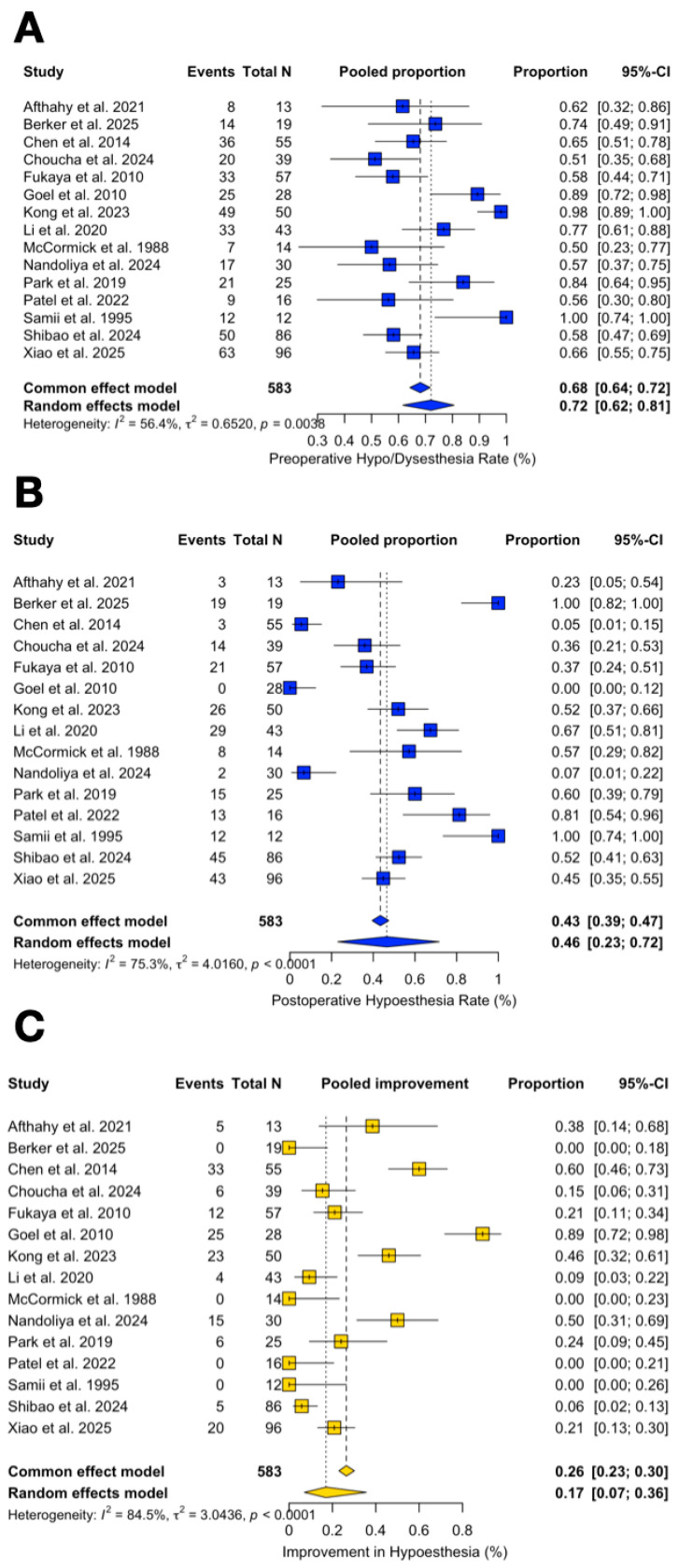
Forest plot showing the preoperative (**A**) and postoperative (**B**) trigeminal impairment rate across studies included and its improvement rate (**C**) [3,4,6,11,13,14,15,16,17,18,19,20,21,22,23]. Blue refers to trigeminal impairment. Yellow refers to improvement rate. The overall meta-analysed measure of effect is represented on the plot as a dashed vertical line. Squares represent individual study effect sizes (with relative solid lines representing confidence intervals). Diamonds summarize the overall pooled effect and its confidence interval.

Improvement rates of trigeminal symptoms (delta improved) were 17.0% (95% CI 7.1–35.6%; I^2^ = 84.5%), while their worsening accounted for 0.07% of cases (95% CI 0–6.5%; I^2^ = 0%) (Figure 8A–C).

Meta-regression revealed that, compared to Samii type A, improvement was significantly more likely in tumours with higher proportions of Samii type B (β = +6.36, *p* = 0.007) and type D (β = +4.70, *p* < 0.001), while no significant difference was observed for type C tumours (*p* = 0.37) (Supplementary Tables S3 and S4).

No significant relationship was observed between year of surgery and improvement rates (β = +0.003, *p* = 0.83).

Although a full model including endoscopic, RSA, and M-AL-Apr was fitted, multicollinearity precluded reliable interpretation of individual surgical approaches (Supplementary Table S5).

### 3.5. Complications

The pooled rate of complications was 11.6% (95% CI 6.7–19.4%; I^2^ = 32.7%). Meta-regression showed an increasing trend in complications over time (β = +0.041, 95% CI +0.001 to +0.089, *p* = 0.066), corresponding to an estimated +4.2% relative increase per year.

**Figure 9:**
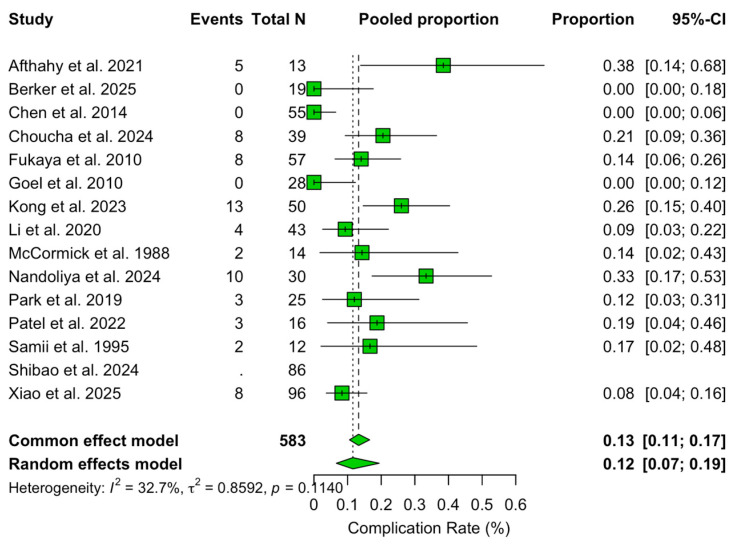
Forest plot showing the complication rate across studies included [3,4,6,11,13,14,15,16,17,18,19,20,21,22,23]. Green refers to complication rate. The overall meta-analysed measure of effect is represented on the plot as a dashed vertical line. Squares represent individual study effect sizes (with relative solid lines representing confidence intervals). Diamonds summarize the overall pooled effect and its confidence interval.

In multivariable analysis, higher proportions of Samii type A (β = +103.04, *p* = 0.004), type C (β = +112.69, *p* = 0.002), and type D (β = +96.04, *p* = 0.007) tumours were independently associated with increased complication rates. No surgical approach remained independently associated with complication rates after adjustment (Figure 9).

The studies included did not report any cases of mortality.

A breakdown of major postoperative complications is reported in Table 3.

### 3.6. Quality Assessment

Using the JBI checklist for case series, 15 studies were assessed as having a low risk of bias and were therefore included. One study was excluded due to a high risk of bias [24] (Table 4).

## 4. Discussion

This meta-analysis provides a comprehensive synthesis of surgical outcomes in trigeminal schwannoma resection, drawing on over 580 cases reported in contemporary series. It elucidates evolving operative strategies, predictors of oncological and functional outcomes, and the delicate balance between efficacy and safety in this rare disease [11,16].

A clear temporal trend toward endoscopic techniques was observed, mirroring the integration of ETOAs into modern skull base surgery. Most frequently employed for Samii type A tumours, typically those involving the middle cranial fossa and lateral skull base, ETOAs provide direct access to regions such as the lateral cavernous sinus and Meckel’s cave with minimal brain retraction. Their use in type B tumours remains limited due to posterior fossa confinement; RCA remains the mainstay. For dumbbell-shaped type C tumours and extracranial-extending type D tumours, ETOAs may be selectively combined with other routes to enhance access while minimizing morbidity. These nuanced applications underscore the importance of tailoring surgical strategy to tumour anatomy. More broadly, this evolution reflects not only a refined surgical philosophy centred on anatomical preservation and functional protection, but also the growing adoption of less invasive approaches made possible by advances in endoscopic technology without compromising oncological control [11,25,26].

The stratified meta-regression identified a temporal decrease in GTR for Samii type C tumours alone, while resection rates for types A, B, and D remained stable. While no significant change occurred for Samii types B and D, and only marginal changes were seen in type A, a marked and statistically significant decline in GTR was observed in type C tumours. This trend likely reflects the increasing proportion of anatomically complex cases in recent series, rather than a general reduction in surgical ambition (see Figure 6). Concurrently, advancements in adjuvant therapies have encouraged a paradigm shift: preserving function may at times take precedence over radical resection, particularly in high-risk cases [6,17,27,28]. Within this framework, STR has gained broader acceptance. The availability of stereotactic radiosurgery (SRS) as an effective adjuvant tool has enabled deliberate conservative resections in select cases, allowing for excellent disease control while mitigating neurological risk. This strategy exemplifies an individualized, multidisciplinary approach to TS management [2].

Surgical treatment remains highly effective in alleviating trigeminal dysfunction. Improvement in pre-existing symptoms was frequently achieved, underscoring the potential for surgery to offer both oncological and symptomatic benefits. Yet, tumour type continues to shape these outcomes. Specifically, higher proportions of Samii type B and D tumours were significantly associated with improved outcomes, whereas type A and especially type C tumours, often more invasive or anatomically complex, were less likely to demonstrate postoperative recovery. These results reinforce the primacy of tumour-specific planning in predicting neurological improvement, independent of surgical era or technique [6,17,27,29,30].

Interestingly, we observed a temporal rise in reported complications, despite the increasing adoption of minimally invasive approaches. This paradox is best explained by the concurrent increase in anatomically complex tumours, again underscoring tumour type as the key driver of morbidity. Importantly, the surgical approach per se was not independently associated with complication risk. This reinforces that approach selection should be dictated by anatomical suitability rather than a priori assumptions about safety profiles [6,11,17,19]. Notably, when specific attention was given to complications associated with individual surgical approaches, an increased incidence of oculomotor impairment was observed with the use of the ETOA, and a higher rate of CSF leakage was noted with the EEA. Conversely, endoscopic techniques were associated with a reduced prevalence of brain retraction-related complications compared to traditional open approaches [6,17,20].

### Limitations

This study has several limitations. First, the included literature consisted predominantly of retrospective observational series, carrying inherent risks of selection bias and heterogeneity in reporting. Although we applied rigorous eligibility criteria and conducted meta-regression analyses to explore sources of heterogeneity, residual confounding cannot be excluded. Second, the lack of direct comparative studies between surgical approaches precluded the ability to perform pairwise comparisons with relative risks; as such, our inferences rely on meta-regression, which, while informative, does not replace direct comparative evidence. Third, the classification of surgical approaches and tumour types, although standardised for analysis, may vary across centres and publications. Fourth, the progressive incorporation of adjunctive therapies over time could influence surgical decision-making and outcomes but was not consistently reported across studies. Fifth, the extreme variation in follow-up durations across studies and the lack of standardised timing for postoperative trigeminal impairment assessments limit the ability to differentiate between early transient deficits and long-term functional outcomes. Sixth, due to the highly specialized nature of this pathology, additional sources such as select book chapters are available; however, these were excluded from the analysis owing to the absence of data specifically pertinent to the objectives of this meta-regression [31,32]. Finally, the low event rate for certain outcomes, particularly complication subtypes, limited the possibility to analyse them systematically.

## 5. Conclusions

This meta-analysis delineates evolving trends and key predictors in the surgical management of trigeminal schwannomas. While the adoption of minimally invasive approaches has expanded, the apparent decline in resection rates and rise in complications reflect an increasing anatomical complexity in modern surgical series. Tumour type remains a pivotal determinant of resection extent, symptomatic recovery, and complication risk, underscoring the need for personalized, anatomy-guided surgical planning. These findings may inform future refinements in surgical decision-making and support the ongoing evolution of patient-centred care in skull-base surgery.

## Figures and Tables

**Table 1:** General data of the studies included.

Study Features						Approaches						Samii Classification %				Clinical Features %							
Author	Year	Type of Studies	Cases	Surgeries	Mean age	ETOA %	EEA %	M-AL Apr. %	RSA %	Comb./2sps %	TMA %	A	B	C	D	Pre Trig. Imp.	Post Trig. Imp.	CSF Leak	Other Compl.	Recurrence	GTR	NTR	STR
Aftahy et al. [4]	2021	RS	13	13	57.50	0	15.38	46.15	23.08	15.38	0	46.15	15.38	30.77	7.69	61.54	23.08	0	38.46	0	76.92	23.08	7.69
Berker et al. [13]	2025	RS	19	19	37.5	0	0	68.42	31.58	0	0	26.32	5.26	68.42	0	73.68	100	0	0	0	84.21	10.53	5.26
Chen et al. [14]	2014	RS	55	55	36	0	0	80	18.18	1.82	0	23.64	18.18	38.18	20	65.45	5.45	0	0	1.82	69.09	23.64	7.27
Choucha et al. [15]	2024	RS	39	39	42	0	15.38	69.23	15.38	0	0	15.38	5.13	46.15	33.33	51.28	35.90	7.69	20.51	7.69	33.33	10.26	56.41
Fukaya et al. [3]	2010	RS	57	57	42.8	0	0	100	0	0	0	14.04	21.05	57.89	12.28	57.89	36.84	3.51	14.04	8.77	80.70	3.51	15.79
Goel et al. [16]	2010	RS	28	28		0	0	100	0	0	0	0.00	0	0	100	89.29	0	0	0	7.14	71.43	0	28.57
Kong et al. [17]	2023	RS	50	50	46.9	100	0	0	0	0	0	34.00	0	40	26	98	52	0	26	0	70	18	8
Li et al. [18]	2020	RS	43	43	45.3	0	13.95	60.47	13.95	11.63	0	18.60	13.95	44.19	23.26	76.74	67.44	0	9.30	2.33	90.70	6.98	2.33
McCormick et al. [19]	1988	RS	14	14	40	0	0	50	35.71	14.29	0	57.14	35.71	0	7.14	50	57.14	14.29	14.29	28.57	85.71	14.29	0
Nandoliya et al. [20]	2024	RS	30	30	43	0	46.67	30	3.33	20	0	20.00	10	50	20	56.67	6.67	6.67	33.33	13.33	53.33	46.67	0
Park et al. [6]	2019	RS	25	25	48.8	44	52	0	0	4	0	36.00	0	32	32	84	60	0	12	4	48	28	24
Patel et al. [23]	2022	RS	16	16	44	0	81.25	0	0	0	18.75	37.50	0	0	62.50	56.25	81.25	37.50	18.75	6.25	50	25	25
Samii et al. [11]	1995	RS	12	12	44	0	0	50	8.33	41.67	0	41.67	8.33	41.67	8.33	100	100	16.67	16.67	16.67	83.33	16.67	0
Shibao et al. [21]	2024	RS	86	86	45	0	2.33	96.51	1.16	0	0	16.28	19.77	40.70	23.26	58.14	52.33	0	0	2.33	74.42	25.58	0
Xiao et al. [22]	2025	RS	96	96	46.3	0	0	70.83	28.13	1.04	0	15.63	8.33	55.21	20.83	65.63	44.79	1.04	8.33	1.04	91.67	5.21	3.13

RS, retrospective series; ETOA, endoscopic transorbital approach; EEA, endoscopic endonasal approach; M-AL-Apr, microsurgical antero-lateral approaches; RSA, retrosigmoid approach; Comb./2sps, combined approach or two-stage surgery; TMA, trans-maxillary approach; Pre Trig. Imp., preoperative trigeminal impairment; Post Trig. Imp., postoperative trigeminal impairment; Compl., complications; GTR, gross-total resection; NTR, near-total resection; STR, subtotal resection.

**Table 2:** Surgical approach distribution by Samii tumour type.

Author	Samii Type	Number of Patients	ETOA	EEA	M-AL Approaches	RSA	Comb./2-steps	TMA
Aftahy et al., 2021 [4]	A	6	0	1	5	0	0	0
Chen et al., 2014 [14]	A	13	0	0	13	0	0	0
Choucha et al., 2024 [15]	A	6	0	2	4	0	0	0
Fukaya et al., 2010 [3]	A	8	0	0	8	0	0	0
Goel et al., 2010 [16]	A	0	0	0	0	0	0	0
Kong et al., 2023 [17]	A	17	17	0	0	0	0	0
Li et al., 2020 [18]	A	8	0	0	8	0	0	0
McCormick et al., 1988 [19]	A	8	0	0	6	0	2	0
Park et al., 2019 [6]	A	9	5	4	0	0	0	0
Patel et al., 2022 [23]	A	6	0	6	0	0	0	0
Samii et al., 1995 [11]	A	5	0	0	5	0	0	0
Aftahy et al., 2021 [4]	B	2	0	0	0	2	0	0
Chen et al., 2014 [14]	B	10	0	0	0	10	0	0
Choucha et al., 2024 [15]	B	2	0	1	0	1	0	0
Fukaya et al., 2010 [3]	B	12	0	0	11	1	0	0
Goel et al., 2010 [16]	B	0	0	0	0	0	0	0
Kong et al., 2023 [17]	B	0	0	0	0	0	0	0
Li et al., 2020 [18]	B	6	0	0	0	6	0	0
McCormick et al., 1988 [19]	B	5	0	0	0	5	0	0
Park et al., 2019 [6]	B	0	0	0	0	0	0	0
Patel et al., 2022 [23]	B	0	0	0	0	0	0	0
Samii et al., 1995 [11]	B	1	0	0	0	1	0	0
Aftahy et al., 2021 [4]	C	4	0	0	1	2	1	0
Chen et al., 2014 [14]	C	21	0	0	20	0	1	0
Choucha et al., 2024 [15]	C	18	0	0	13	5	0	0
Fukaya et al., 2010 [3]	C	33	0	0	33	0	0	0
Goel et al., 2010 [16]	C	0	0	0	0	0	0	0
Kong et al., 2023 [17]	C	20	20	0	0	0	0	0
Li et al., 2020 [18]	C	19	0	0	17	0	2	0
McCormick et al., 1988 [19]	C	0	0	0	0	0	0	0
Park et al., 2019 [6]	C	8	4	3	0	0	1	0
Patel et al., 2022 [23]	C	0	0	0	0	0	0	0
Samii et al., 1995 [11]	C	5	0	0	0	0	5	0
Aftahy et al., 2021 [4]	D	1	0	1	0	0	0	0
Chen et al., 2014 [14]	D	11	0	0	11	0	0	0
Choucha et al., 2024 [15]	D	13	0	4	9	0	0	0
Fukaya et al., 2010 [3]	D	7	0	0	6	0	1	0
Goel et al., 2010 [16]	D	28	0	0	28	0	0	0
Kong et al., 2023 [17]	D	13	13	0	0	0	0	0
Li et al., 2020 [18]	D	10	0	6	1	0	3	0
McCormick et al., 1988 [19]	D	1	0	0	1	0	0	0
Park et al., 2019 [6]	D	8	2	6	0	0	0	0
Patel et al., 2022 [23]	D	10	0	7	0	0	0	3
Samii et al., 1995 [11]	D	1	0	0	1	0	0	0

**Table 3:** Major postoperative complications.

Author	Year	CSF Leak Overall %	CSF Leak in EEA %	CSF Leak in Other Non-Endoscopic Approaches %	Oculomotor Dysfunction %	Facial Palsy %	Retraction Related Complications %	Infections %
McCormick et al. [19]	1988	14.29		100			7.14	7.14
Samii et al. [11]	1995	16.67		100		16.67		
Fukaya et al. [3]	2010	3.51		100	14.04			
Goel et al. [16]	2010							
Chen et al. [14]	2014							
Park et al. [6]	2019				4		4	4
Li et al. [18]	2020				2.33	2.33		4.65
Aftahy et al. [4]	2021				15.38	15.38	7.69	
Patel et al. [23]	2022	37.50	83.33	16.67	12.50		6.25	
Kong et al. [17]	2023				22			4
Choucha et al. [15]	2024	7.69		100			10.26	10.26
Nandoliya et al. [20]	2024	6.67		100	26.67		3.33	3.33
Shibao et al. [21]	2024							
Berker et al. [13]	2025							
Xiao et al. [22]	2025	1.04		100	2.08	2.08	4.17	

**Table 4:** Risk of bias—JBI checklist for case series.

Study	Year	1. Were There Clear Criteria for Inclusion in the Case Series?	2. Was the Condition Measured in a Standard, Reliable Way for All Participants Included in the Case Series?	3. Were Valid Methods Used for Identification of the Condition for All Participants Included in the Case Series?	4. Did the Case Series Have Consecutive Inclusion of Participants?	5. Did the Case Series Have Complete Inclusion of Participants?	6. Was There Clear Reporting of the Demographics of the Participants in the Study?	7. Was There Clear Reporting of Clinical Information of the Participants?	8. Were the Outcomes or Follow-up Results of Cases Clearly Reported?	9. Was There Clear Reporting of the Presenting site(s)/Clinic(s) Demographic Information?	10. Was Statistical Analysis Appropriate?	Include
Aftahy et al. [4]	2021	✕	✓	✓	✓	✓	✓	✓	✓	✓	✓	✓
Berker et al. [13]	2025	✓	✓	✓	✓	✓	✓	✓	✓	✓	✓	✓
Bordi et al.	1989	✕	✓	✓	✓	✓	✕	✕	✕	✓	✓	✕
Chen et al. [14]	2014	✓	✓	✓	✓	✓	✓	✓	✓	✓	✓	✓
Choucha et al. [15]	2024	✓	✓	✓	✓	✓	✓	✓	✓	✓	✓	✓
Fukaya et al. [3]	2010	✓	✓	✓	✓	✓	✓	✓	✓	✓	✓	✓
Goel et al. [16]	2010	✕	✓	✓	✓	✓	✕	✓	✓	✓	✓	✓
Kong et al. [17]	2023	✓	✓	✓	✓	✓	✓	✓	✓	✓	✓	✓
Li et al. [18]	2020	✓	✓	✓	✓	✓	✕	✓	✓	✓	✓	✓
McCormick et al. [19]	1988	✕	✓	✓	✓	✓	✕	✓	✓	✕	✓	✓
Nandoliya et al. [20]	2024	✓	✓	✓	✓	✓	✓	✓	✓	✓	✓	✓
Park et al. [6]	2019	✓	✓	✓	✓	✓	✓	✓	✓	✓	✓	✓
Patel et al. [23]	2022	✓	✓	✓	✓	✓	✓	✓	✓	✓	✓	✓
Samii et al. [11]	1995	✓	✓	✓	✓	✓	✓	✓	✓	✓	✕	✓
Shibao et al. [21]	2024	✓	✓	✓	✓	✓	✓	✓	✓	✓	✓	✓
Xiao et al. [22]	2025	✓	✓	✓	✓	✓	✓	✓	✓	✓	✓	✓

✕ stands for no. ✓ stands for yes.

## Data Availability

The data presented in this study are available on request from the corresponding author.

## Supplementary Materials

The following supporting information can be downloaded at: https://www.mdpi.com/article/10.3390/jcm14134488/s1, Table S1: Multivariable Meta-Regression Results for GTR; Table S2: Meta-regression by association of GTR and Samii Type over Year Quartiles; Table S3: Multivariable Model for Trigeminal Symptom Improvement; Table S4: Predictors of Trigeminal Function Improvement; Table S5: Meta-regression: Functional Improvement by Surgical Approach.

## Abbreviations

CI	Confidence interval
Comb./2sps	Combined approach or two-step surgical strategy
EEA	Endoscopic endonasal approach
ETOA	Endoscopic transorbital approach
FOZ	Fronto-orbitozygomatic
GLM	General linear model
GTR	Gross-total resection
M-AL-Appr	Microsurgical antero-lateral approaches
NTR	Near-total resection
RSA	Retrosigmoid approach
SRS	Stereotactic radiosurgery
STR	Subtotal resection
TMA	Trans-maxillary approach
TS	Trigeminal schwannoma
