# Supplementary material for: Evolution of Surgical Approaches for Trigeminal Schwannomas: A Meta-Regression Analysis from Past to Present
Source: J Clin Med. 2025 Jun 25;14(13):4488. doi: 10.3390/jcm14134488 (PMC12249520; doi:10.3390/jcm14134488)
Supplement: Supplementary file 1 [file jcm-14-04488-s001.zip › jcm-3705191-supplementary.pdf]

Supplementary Table 1. Multivariable Meta-Regression Results for GTR

Predictor	β Coefficient	95% CI	p-value
Endoscopic approach (%)	−1.99	[−10.80, 6.82]	0.661
Transpetrosal approach (%)	−2.95	[−11.65, 5.76]	0.454
Retrosigmoid approach (%)	−13.66	[−19.53, −7.80]	<0.001
Samii type B (%)*	−42.33	[−74.28, −10.37]	0.010
Samii type C (%)*	−59.75	[−97.60, −21.91]	0.002
Samii type D (%)*	−60.30	[−98.00, −22.60]	0.002
Year of publication	−0.003	[−0.022, 0.016]	0.763

$p < 0.001$, likelihood ratio test; * Reference group Samii type A.

Supplementary Table 2 – Meta-regression by association of GTR and Samii Type over Year Quartiles

Samii Type*	Year Quartile	Estimate	Std. Error	z value	p-value
A	(2011,2021]	-1.7848	0.6751	-2.644	0.0082
A	(2021,2024]	-0.5008	0.8537	-0.587	0.55747
C	(2011,2021]	-0.1315	0.4162	-0.316	0.75213
C	(2021,2024]	-1.2731	0.3973	-3.204	0.00135
D	(2021,2024]	-0.9163	0.6519	-1.406	0.1599

*Dependent variable: GTR; Samii Type A: Likelihood ratio test $\chi^2(2) = 8.512$, p = 0.0142; Samii Type B: Not enough quartile-level data to compute; Samii Type C: Likelihood ratio test $\chi^2(2) = 17.176$, p = 0.00019; Samii Type D: Likelihood ratio test $\chi^2(1) = 1.999$, p = 0.1574

Supplementary Table 3 – Multivariable Model for Trigeminal Symptom Improvement

Variable	Estimate (β)	Model
Intercept	-47.4641	Multivariable (Improvement of trigeminal symptoms)
Samii type A (%)	-7.2225	Multivariable (Improvement of trigeminal symptoms)
Samii type C (%)	-5.2955	Multivariable (Improvement of trigeminal symptoms)
Samii type D (%)	-2.0715	Multivariable (Improvement of trigeminal symptoms)
Year	0.0256	Multivariable (Improvement of trigeminal symptoms)

Supplementary Table 4 – Predictors of Trigeminal Function Improvement

Coefficient	Estimate	Std. Error	z value	p value
(Intercept)	-2.708	1.412	-1.9178	0.0551
pct_samii_B	4.631	1.776	2.6075	0.0091
pct_samii_C	1.783	1.123	1.5877	0.1124
pct_samii_D	4.781	1.17	4.0863	0.0

*Reference group Samii Type A

Supplementary Table 5 – Meta-regression: Functional Improvement by Surgical Approach

Predictor	Estimate	Std. Error	z value	p value
Intercept	-94.17	8851.28	-0.011	0.992
pct_endoscopic	93.72	8851.28	0.011	0.992
pct_RSA	91.73	8851.28	0.01	0.992
pct_transpetrosal	94.52	8851.28	0.011	0.991
